# Multisite spontaneous hematomas and bleeding in critically ill Chinese patients with COVID-19: two case reports and a literature review

**DOI:** 10.1186/s12879-024-09012-w

**Published:** 2024-01-18

**Authors:** Sha Sha, Sun Qi, Shi Qindong

**Affiliations:** 1 Department of Critical Care Medicine, the First Affiliated Hospital of Xi’an Jiaotong University, Xi’an, Shaanxi China; 2 Department of Intensive Care Medicine, the Power Central Hospital of Genertec Guozhong Healthcare Limited Company, Xi’an, Shaanxi 710032 China

**Keywords:** COVID-19, Anticoagulation, Heparins, Hemorrhage, Multisite hematomas, Case report

## Abstract

**Background:**

Anticoagulation is recommended as a standardized therapy for COVID-19 patients according to the WHO guidelines. However, bleeding events have also been reported. Hemorrhage or hematoma was observed in sites including the retroperitoneum, brain, alimentary tract, muscles, and soft tissues. Reduction or suspension of anticoagulants is a common intervention. Transfusion, endoscopic hemostasis, and vascular interventional therapy have been used to improve the condition.

**Case presentation:**

In this article, we present two cases of concurrent multisite hematomas and bleeding at other sites in patients with SARS-CoV-2 infection. Both patients were treated with heparins and experienced bleeding after the anticoagulation therapy. Both patients were older with more than two comorbidities, and critical COVID-19. Laboratory tests revealed a considerable decrease in hemoglobin levels and alterations in the coagulation system. In the first patient, the main intervention was embolization using angiography. However, we only adjusted the anticoagulation strategy in the second case. The first patient recovered and was discharged; however, the second died of other causes. This study provides a retrospective review of typical hemorrhagic cases during anticoagulation in COVID-19 patients over the course of four years. A relatively comprehensive search was performed in Pubmed by constructing MeSH subject terms on limiting the search period and specific contents. It summarizes and synthesizes the research related to heparins and other novel anticoagulants in the context of COVID-19 from the onset of the pandemic to the present disseminated phase. This study aimed to offer valuable insights and reference points for developing anticoagulation treatment strategies for patients with COVID-19.

**Conclusions:**

Anticoagulation is a crucial treatment option for patients with COVID-19. The difference in anticoagulant effects is related to the severity of COVID-19. Nafamostat can reduce thrombosis in the extracorporeal circuits in critically ill patients with COVID-19. The efficacy and safety of novel anticoagulants require further clinical data. Routine bedside assessments and real-time laboratory monitoring are essential for early identification of bleeding events during anticoagulant therapy and administering intervention.

## Background

Nearly four years have passed since the start of COVID-19 pandemic. Omicron is currently the main infectious strain with the lowest mortality rate. However, during the Delta pandemic, the global excess mortality was estimated at 14.9 million in 2020 and 2021. In 2021, the proportion of critically ill patients with COVID-19 reached 3.32% in China. Severe coagulation disorder can be present in almost 20% of patients with COVID-19 and likely occurs in the most severe and critically ill COVID-19 cases [1]. According to autopsy studies, thrombosis can be found at many sites [2, 3]. Increased D-dimer levels have also been observed in critically ill patients with COVID-19 [4]. Previous studies have demonstrated that the mechanism associated with COVID-19 related coagulation abnormalities involve vascular endothelial cell injury, abnormal fibrinolytic pathways induced by cytokine storms, coagulation cascade hyperactivation, downregulation of activated protein C, and inhibition of fibrinolysis. These processes exacerbate the formation of pulmonary and intravascular macrothrombi, resulting in an increased proportion of critical cases and higher mortality [5, 6]. Anticoagulant therapy has proven to be effective in reducing COVID-19 mortality, especially in critically ill patients [7–9]. Both guidelines recommend a standard anticoagulation strategy according to the different conditions and types of COVID-19 [10, 11].

From the end of 2021 to the beginning of 2022, COVID-19 spread to large areas of Xi’an City in China. A total of 55 patients were diagnosed with severe or critical COVID-19. All patients received individualized anticoagulation therapy. Among these patients, only four progressed with bleeding events and two had multisite hematomas along with bleeding in other organs. One patient recovered and was discharged, while the other died of comorbidities.

Below, we present these two cases and provide a retrospective review of typical hemorrhagic cases during anticoagulation in patients with COVID-19 over the course of four years. We also summarized studies on heparins and other novel anticoagulants. This study aimed to offer a reference for anticoagulation strategies among patients with COVID-19.

### Case presentation

#### Case no.1

A 73-year-old man with a long history of hypertension, diabetic nephropathy, and coronary disease, who had never accepted the COVID-19 vaccine, was diagnosed with COVID-19 on December 21, 2021. A real-time polymerase chain reaction results (RT-PCR) for SARS-CoV-2 were negative on January 1, 2022. However, he was admitted to the ICU and required advanced life support due to a series of severe complications of the SARS-CoV-2 infection and existing chronic comorbidities. The laboratory test results are presented in Table 1.

**Table 1:** Table 1

	case1	case2
**General information**		
age	73	90
sex	Male	Male
vaccination status	NV	NV
preexisting condition	CKD stage 4 Hypertension Diabetes Coronary Disease	Hypertension Atrial Fibrillation
severity of COVID-19	critical	critical
BMI(kg/m^2^)	28.3	20.2
**Onset and Progress**		
Date of onset	2021/12/21	2021/12/17
Date of entering ICU	2022/1/4	2022/1/1
Duration between onset and entering ICU(d)	13	15
Days of invasive mechanical ventilation(d)	9	>28
Days of renal replacement therapy(d)	>28	**/**
**Anticoagulation Therapy**		
Date of starting anticoagulants	2022/1/4	2022/1/1
Date of hematoma or hemorrhage	2022/1/21	2022/2/7
Duration between starting anticoagulation and first bleeding event(d)	18	37
Initial dose	LMWHs (2500 IU q12h)	LMWHs (2500 IU q12h)
Dosage at hematoma or bleeding	LMWHs (7500 IU q12h)	Nadroparin Calcium (3264 IU qd)
Sites of hematoma or bleeding	hepatic hemorrhage retroperitoneal hematoma(right) iliopsoas hematoma(right) gastrointestinal tract	scapula muscular hematoma(left) retroperitoneal hematoma(left) gastrointestinal tract
Interventions	1.TAE 2.anticoagulation suspended 3.nafamostat mesilate	anticoagulation suspended
Cumulative days of anticoagulation therapy(d)	28	29
Cumulative days of suspending anticoagulation(d)	11	6
**Transfusion**		
RBC(u)	16	16
Platelet(u)	20	/
Plasma(ml)	1200	800
**Outcomes**	**Alive**	**Died**

NV = Not Vaccinated; BMI = Body Mass Index; LMWHs = Low-Molecular-Wight Heparin Sodium; TAE = Transarterial Embolization; RBC = Red Blood Cell; CKD = Chronic Kidney Disease

On January 4th, as chronic diabetic nephropathy progressed to acute renal injury, and oxygenation worsened, the patient received mechanical ventilation (MV) and continuous renal replacement therapy (CRRT). Considering the potential for microthrombosis and the need for blood purification therapy, clinicians initiated anticoagulation therapy with a therapeutic dose of Low-Molecular-Weight Heparin sodium (LMWHs). The primary dosage was 2500 IU and the interval was q12h. Regional citrate anticoagulation was combined with CRRT. The LWMHs dosage was adjusted to 5000 IU q12h from January 9th and was finally regulated to 7500 IU q12h from January 11th, according to laboratory test results.

**Fig. 1:**
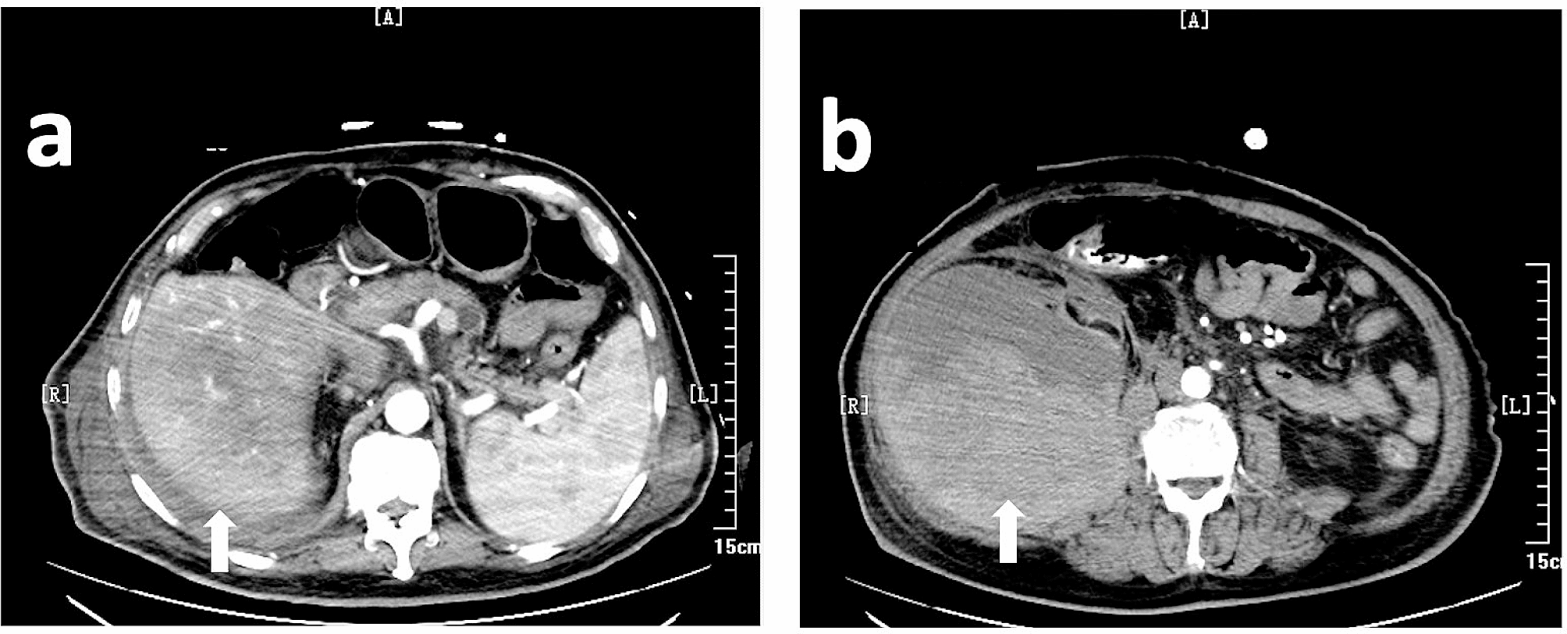
The laboratory test results of patients from admission to discharge or death. The upper two charts represent the first patient, and the lower chart represents the second patient

**Fig. 2:**
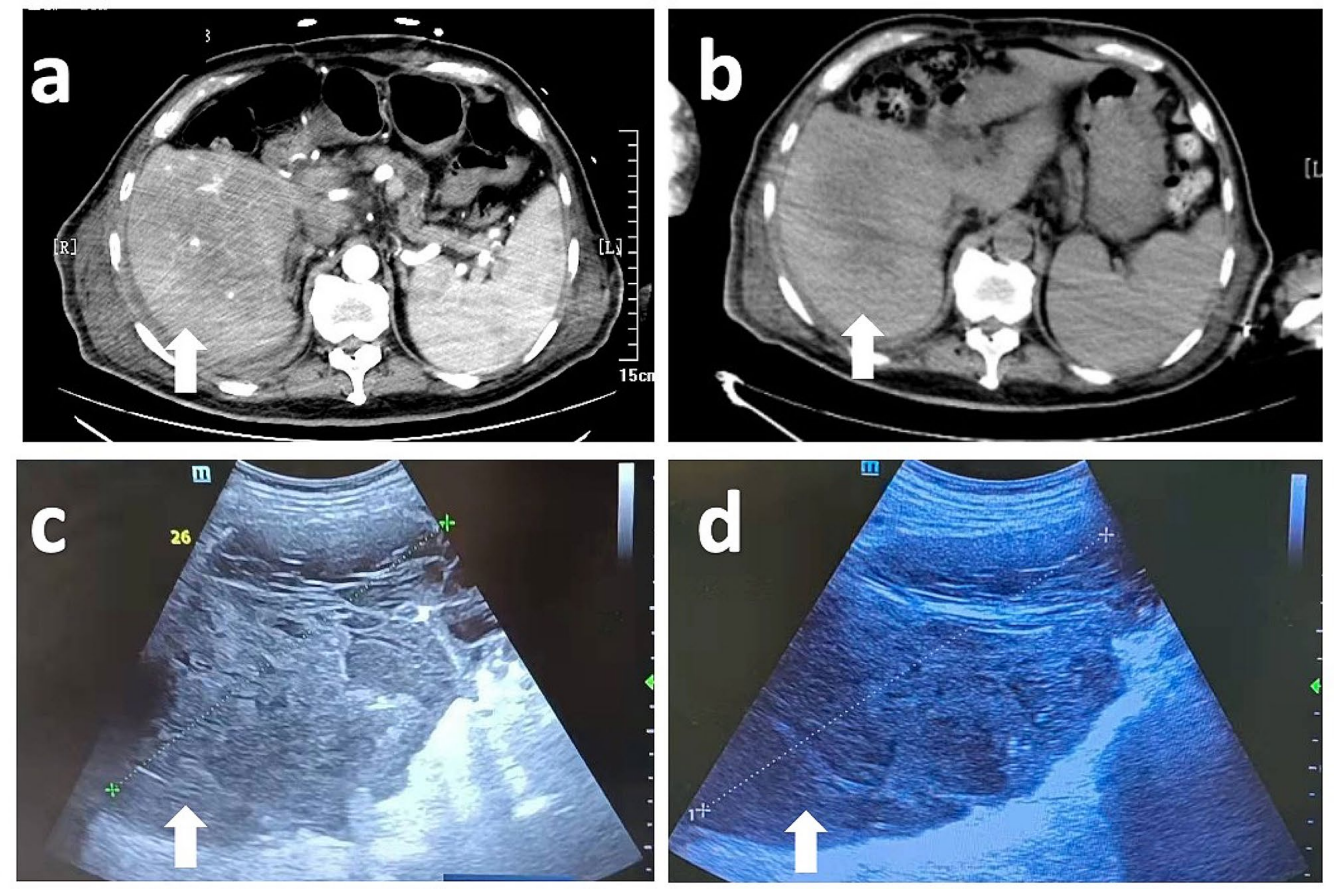
Computed Tomography images of the patients’ hematomas. This image shows the 14.5*11*11.3 cm hepatic hematoma and the 24.7*12.6*12.6 cm retroperitoneal hematoma (white arrows)

However, on January 20, 2022, his blood pressure dropped to 80/50 mmHg (with norepinephrine), and the heart rate rose to at least 140 bpm on a real-time monitor. Before the patient experienced a disturbance of consciousness, the nurse found early symptoms and signs of shock, including irritability, thirst, and a weak pulse. Soon after, he lost consciousness, the skin and mucus turned pale, and the extremities felt cold. Laboratory test results are seen in Fig. 1. The patient then underwent a contrast-enhanced whole body CT scan, revealing hepatic hemorrhage, right spontaneous retroperitoneal hematoma, and iliopsoas hematoma. (Fig. 2)

**Fig. 3:**
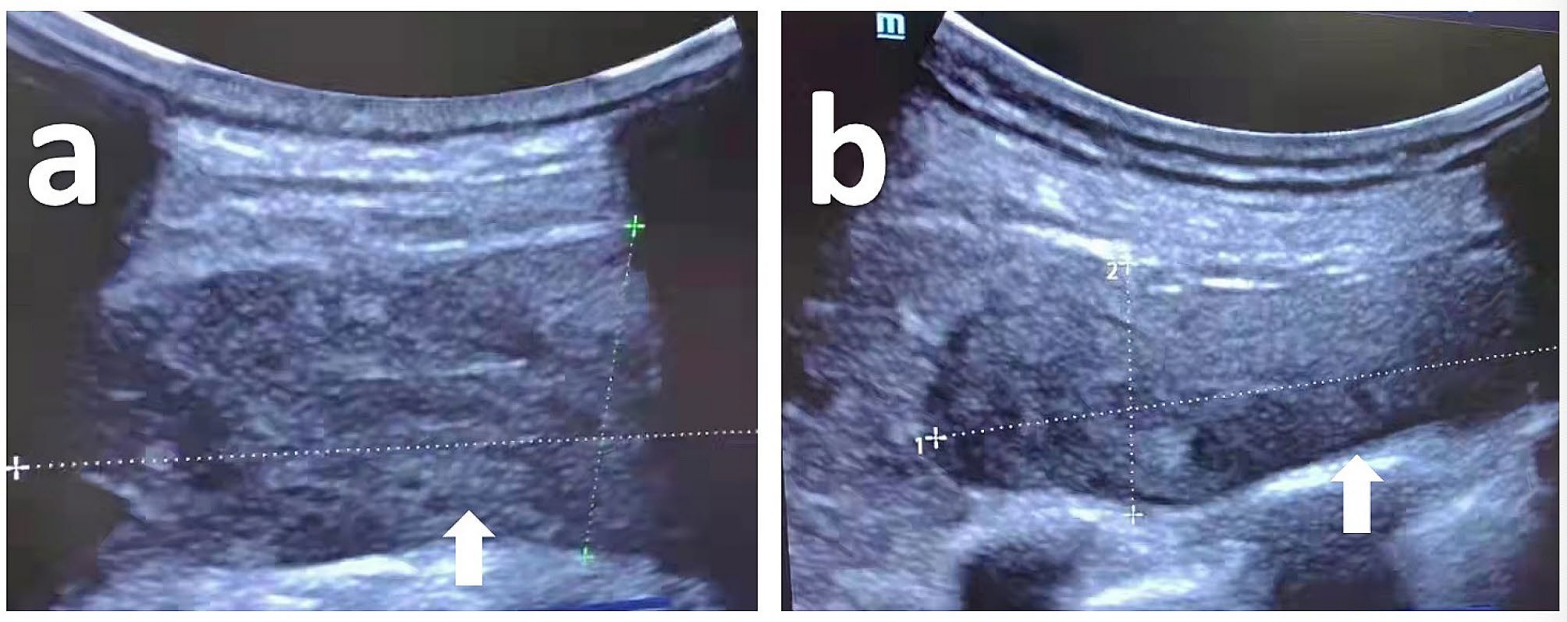
Computed Tomography and ultrasound images of the patients’ hematomas. The image of the hepatic hematoma taken on Jan.13th, and b shows the area diminished on Feb.3rd (white arrows). The point-of-care ultrasound images shows the changes in retroperitoneal hematoma.(c: 24.7*12.6*12.6 cm; d: 15.2*9.6*8.8 cm, white arrows)

On January 22nd, as the hemodynamics were steady, he underwent digital subtraction angiography and interventional embolization treatment. Anticoagulants were discontinued to prevent rebleeding. Owing to persistent renal insufficiency, an anticoagulant (nadroparin calcium, 4100 IU) only was administered on the day of intermittent kidney replacement therapy from January 29th. The cumulative discontinuation days of LMWHs were 11. On February 3rd, point-of-care ultrasound showed that the area of fluid within the hematoma was reduced compared to previously, considering thrombus mechanization (Fig. 3). The laboratory test results are also presented in Fig. 1.

Overall, the patient received a total volume of 16 u red blood cell transfusion, and the plasma transfusion volume was 1200 ml and the platelet volume was 20 u. On February 18th, the patient recovered and was discharged. However, he will require kidney replacement therapy for the rest of his life due to his chronic renal insufficiency.

#### Case no.2

The second patient was a 90-year-old male, with chronic hypertension and atrial fibrillation who was receiving warfarin anticoagulant therapy. He had never received the COVID-19 vaccine and was diagnosed with COVID-19 on December 17, 2021. His RT-PCR results were negative on December 31, 2021. Due to severe respiratory symptoms, the patient was admitted to the ICU on January 1, 2022. MV was induced via tracheal intubation. The doctors arranged for daily monitoring of respiratory mechanics and intermittent alveolar lavage under fiberoptic bronchoscopy. Laboratory test results on admission are presented in Fig. 1.

**Fig. 4:**
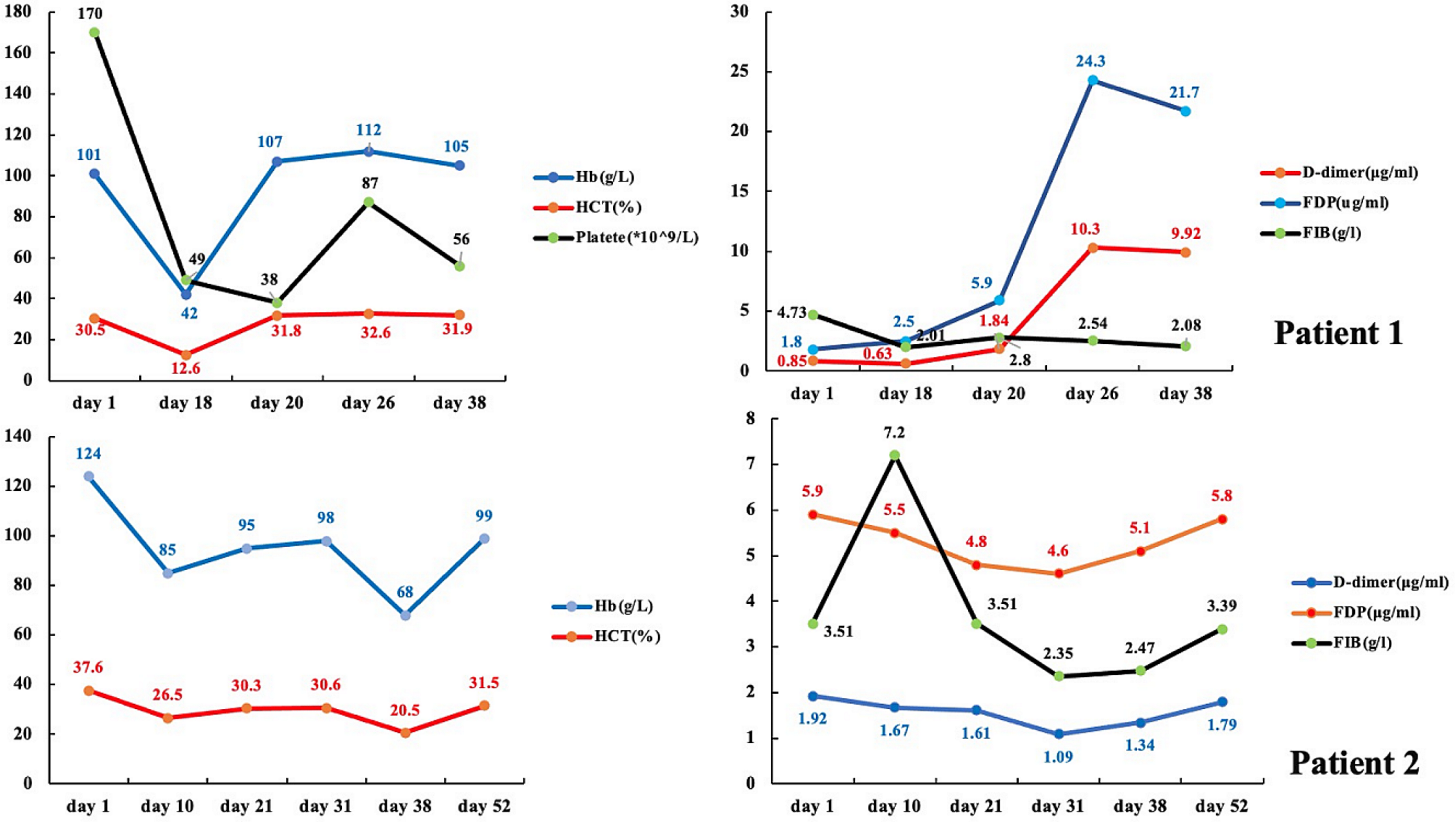
Point-of-care ultrasound images of the patients’ hematomas. This image shows the 8.0*3.3*9.8 cm hematoma under the left scapula (**a**) and the 7.6*3.2 cm hematoma in the left retroperitoneum (**b**) (white arrows)

Anticoagulation therapy was initiated on the patient’s admission to the ICU due to atrial fibrillation and the prevention of microthrombosis for COVID-19. The anticoagulation regimen was LMWHs, 2500 IU, q12h. The routine blood test results were closely monitored. On January 10th, as both the D-dimer and fibrin degradation products (FDP) levels increased, the LMWHs were adjusted to 5000 IU at q12h. Unfortunately, on January 21st, the patient experienced an episode of fibrillation, and D-dimer and FDP levels increased again. Amiodarone was administered to restore the sinus rhythm, and heparin nebulization anticoagulation was added. To prevent thrombosis and reduce the risk of bleeding, the anticoagulation regimen was changed to continuous pumping of nadroparin calcium (3264 IU, qd). During treatment, the patient was closely monitored for coagulation function and other relevant laboratory indices in case of gastrointestinal bleeding. The patient’s occult blood test results remained positive on January 31st. On February 7th, a point-of-care ultrasound examination detected an 8.0*3.3*9.8 cm hematoma in the soft tissue of the left scapula and a 7.6*3.2 cm hematoma in the left retroperitoneum (Fig. 4). The drainage fluid in the gastrointestinal decompression tube turned red, indicating bleeding from the upper alimentary tract.

Therefore, anticoagulation therapy was discontinued immediately, with urgent transfusion of red blood cells and plasma. Six days later, the left subscapular hematoma was reduced to 1.5*1.7 cm, and the left retroperitoneal hematoma had disappeared. During the entire process, the patient received a total volume of 16 u red blood cell transfusion and the plasma transfusion volume was 800 ml. Unfortunately, the patient died from severe respiratory failure.

### Literature review

#### Method

In this literature review, the authors conducted a relatively comprehensive search of Pubmed using MeSH subject terms on COVID-19 and anticoagulation. The inclusion criteria were limited to randomised controlled trials(RCTs) or meta-analyses published between 2020 and 2023, focusing on topics such as heparins, novel anticoagulants, or anticoagulant bleeding complications. Ultimately, 11 RCTs or meta-analyses were included in the literature review. The authors summarised four previously published case reports on anticoagulant dosage and bleeding complications, which also reported possible dose-related haematoma complications.

## Discussion

With the outbreak and pandemic of COVID-19 progressing to the current disseminated stage, anticoagulant therapy has gained prominence as a primary treatment strategy, particularly for patients with severe or critical COVID-19 [11]. Early autopsy studies have found widespread microthrombi and macrothrombi throughout the bodies of patients with COVID-19 [2, 3]. SARS-CoV-2 induces an exacerbated inflammatory response, leading to pulmonary injury encompassing microvascular and endothelial dysfunction, culminating in secondary coagulation abnormalities and thrombus formation. These pathological processes exacerbate the clinical course and increase the mortality rates in patients with COVID-19. Upon heparins binding to the spike protein of SARS-CoV-2, competitively inhibiting viral entry, thereby mitigating potential inflammatory cascades and secondary coagulopathies [12]. The 2020 HESACOVID study demonstrated improvements in P/F ratio, more ventilator-free days, and enhanced survival outcomes with heparin administration [4]. A recent study suggested that heparins might not significantly enhance clinical outcomes in critically ill patients with COVID-19 compared to other anticoagulants or conventional methods [13]. However, its role in reducing thrombotic events in patients with COVID-19 remains unequivocal. A meta-analysis conducted this year further supports the efficacy of heparin in lowering thrombotic risk among patients in the ICU [14].

Hemorrhage is a critical concern in heparin anticoagulation therapy. From 2020 to 2022, clinicians from different countries and areas reported cases of spontaneous retroperitoneal or muscular hematoma with a prophylactic or therapeutic dosage of heparins among COVID-19 patients. These illustrative cases are summarized in Table 2. These findings align with the risk factors observed in our reported cases, demonstrating a predisposition to severe bleeding, including life-threatening hemorrhages, among middle-aged to elderly males with concomitant chronic ailments. The COVID-PACT study underscored that while heparins at full therapeutic doses did not increase in-hospital mortality rates in critically ill COVID-19 patients, hemorrhagic events predominantly manifested as moderate to severe, necessitating transfusion therapy. Given the absence of subgroup analysis differentiating outcomes between UFH and LMWHs in this study, differences in bleeding tendencies attributable to varying heparin-based drugs remain unclear [15]. UFH may confer greater advantages in critically ill COVID-19 patients with concomitant renal insufficiency [16]. Moreover, another randomized controlled trial (RCT) implied that escalated anticoagulation doses may increase the risk of bleeding in critically ill patients, with excessively high anticoagulation dosages increasing the incidence of major hemorrhagic events [17]. Patients with COVID-19 in the ICU may not necessarily derive substantial benefits from aggressive anticoagulation compared to the prophylactic dose [13, 14]. The efficacy of heparins at both prophylactic and therapeutic doses remains contentious for COVID-19 patients in the ICU. Given the heterogeneity of anticoagulation requirements in critically ill COVID-19 patients, determining the optimal dosage poses a greater challenge [18]. Additionally, factors such as heparin-induced thrombocytopenia, prone positioning ventilation, cardioversion, and extracorporeal circulation may augment bleeding risk during heparin anticoagulation in critically ill patients [19–21].

**Table 2:** Table 2

No.	Age(y)	Gender	Preexisting diseases	Anticoagulants	Sites of bleeding	Dosage at bleeding	Interventions	Outcomes
1	86 [22]	female	cardiovascular disease	enoxaparin	hematoma in the left thigh	6000 IU bid	Transfusion	alive
2	81[22]	male	hypertension T2DM IHD	enoxaparin	hematoma in the right thigh	8000 IU bid	TAE transfusion	died
3	69[23]	male	CAD hypertension T2DM	enoxaparin	hematoma in the right psoas muscle	1 mg/kg per day	TAE transfusion	alive
4	62 [24]	male	not mentioned	enoxaparin	left iliopsoas muscle	40 mg per day	TAE transfusion	alive
5	79 [24]	male	not mentioned	UFH	right iliopsoas muscle with retroperitoneal hematoma	not mentioned	TAE transfusion	died
6	57 [25]	male	none	enoxaparin	right retroperitoneal and psoas muscle hematoma	40 mg per day	surgery transfusion	alive

TAE = Transarterial Embolization; UFH = Unfractionated Heparin; IHD = Ischemic Heart Disease; CAD = Coronary Heart Disease; T2DM = Diabetes Mellitus Type 2

Interestingly, despite dynamic adjustments in heparin dosage guided by monitored activated partial thromboplastin time and activated clotting time, one of these two patients exhibited frequent coagulation and thrombus formation in the extracorporeal circuits during CRRT. However, circuit thrombosis and mucosal bleeding diminished with the concomitant use of NM. NM is a novel anticoagulant with a short half-life and is rapidly metabolized in the body. This new anticoagulant has demonstrated favorable anti-inflammatory and anticoagulated qualities, especially in patients with sepsis during blood purification, enhanced survival rates, and reduced bleeding risk [26]. An earlier study showed that NM potently inhibited SARS-CoV-2 spike protein-mediated fusion in a cell fusion assay system and inhibited SARS-CoV-2 infection in vitro in a cell type-dependent manner [27].

Related studies conducted in Japan and Korea suggested that a combination of heparin and NM therapy may be more effective in overcoming COVID-19-related coagulopathy and disseminated intravascular coagulation [28, 29]. A 2022 United Kingdom RCT did not support the efficacy of intravenous NM in critically ill COVID-19 patients [30]. In high-risk bleeding scenarios necessitating support from extracorporeal circulation devices, such as CRRT and extracorporeal membrane oxygenation, NM remains a viable anticoagulation measure to ensure proper circuit operation and minimize bleeding risk.

While NM has demonstrated advantages in extracorporeal anticoagulation in this Xi’an experience, it was administered in combination with heparin rather than independently. The extrapolation of results from RCTs investigating other novel anticoagulants, such as Prostaglandin E1and tissue factor inhibitors, is constrained by factors such as sample size, severity of COVID-19, and different treatment scenarios [18, 31]. Therefore, large-scale studies are required to validate the treatment effects and adverse reactions of these novel anticoagulants.

## Conclusions

Anticoagulant therapy plays a pivotal role in the management of COVID-19 patients. However, the heterogeneity of disease severity in critically ill COVID-19 patients necessitates dose adjustments, with vigilance for bleeding complications, especially fatal hemorrhagic events, in high-risk populations such as elderly males with underlying chronic conditions. Although NM demonstrated extracorporeal anticoagulation advantages in this case report, the evaluation of novel anticoagulants in COVID-19 patients requires robust clinical data. Routine bedside assessments, real-time laboratory monitoring, and the concurrent use of multiple coagulation markers are crucial for identifying early interventions for bleeding events during anticoagulant therapy.

## Data Availability

All data generated or analyzed in this study are included in the published article. The specific laboratory and imaging data are available from the corresponding author upon request.

## Abbreviations

COVID-19: Coronavirus Disease 2019
SARS CoV-2: Severe Acute Respiratory Syndrome Coronavirus 2
MV: Mechanical Ventilation
LMWHs: Low-Molecular-Weight Heparin sodium
FDP: Fibrin Degradation Products
BMI: Body Mass Index
RCT: Randomized Controlled Trial
CKD: Chronic Kidney Disease
UFH: Unfractionated Heparin
IHD: Ischemic Heart Disease
CAD: Coronary Heart Disease
T2DM: Diabetes Mellitus Type 2
NM: Nafamostat mesylate
CRRT: Continuous Renal Replacement Therapy
ECMO: Extracorporeal Membrane Oxygenation

## References

[1] Zhai Z, Li C, Chen Y, Gerotziafas G, Zhang Z, Wan J, Liu P, Elalamy I, Wang C (2020). Prevention Treatment of VTEAwC-ICSG: Prevention and treatment of venous Thromboembolism Associated with Coronavirus Disease 2019 infection: a Consensus Statement before guidelines. Thromb Haemost.

[2] Fox SE, Akmatbekov A, Harbert JL, Li G, Quincy Brown J, Vander Heide RS (2020). Pulmonary and cardiac pathology in African American patients with COVID-19: an autopsy series from New Orleans. Lancet Respir Med.

[3] Middeldorp S, Coppens M, van Haaps TF, Foppen M, Vlaar AP, Muller MCA, Bouman CCS, Beenen LFM, Kootte RS, Heijmans J (2020). Incidence of venous thromboembolism in hospitalized patients with COVID-19. J Thromb Haemost.

[4] Lemos ACB, do Espírito Santo DA, Salvetti MC, Gilio RN, Agra LB, Pazin-Filho A, Miranda CH (2020). Therapeutic versus prophylactic anticoagulation for severe COVID-19: a randomized phase II clinical trial (HESACOVID). Thromb Res.

[5] Li H, Liu L, Zhang D, Xu J, Dai H, Tang N, Su X, Cao B (2020). SARS-CoV-2 and viral sepsis: observations and hypotheses. Lancet.

[6] Ren B, Yan F, Deng Z, Zhang S, Xiao L, Wu M, Cai L (2020). Extremely high incidence of lower extremity deep venous thrombosis in 48 patients with severe COVID-19 in Wuhan. Circulation.

[7] Giossi R, Menichelli D, Pani A, Tratta E, Romandini A, Roncato R, Nani A, Schenardi P, Diani E, Fittipaldo VA (2021). A systematic review and a Meta-analysis comparing prophylactic and therapeutic low Molecular Weight Heparins for Mortality reduction in 32,688 COVID-19 patients. Front Pharmacol.

[8] Paranjpe I, Fuster V, Lala A, Russak AJ, Glicksberg BS, Levin MA, Charney AW, Narula J, Fayad ZA, Bagiella E (2020). Association of Treatment Dose Anticoagulation with In-Hospital survival among hospitalized patients with COVID-19. J Am Coll Cardiol.

[9] Sholzberg M, Tang GH, Rahhal H, AlHamzah M, Kreuziger LB, Ainle FN, Alomran F, Alayed K, Alsheef M, AlSumait F (2021). Effectiveness of therapeutic heparin versus prophylactic heparin on death, mechanical ventilation, or intensive care unit admission in moderately ill patients with covid-19 admitted to hospital: RAPID randomised clinical trial. BMJ.

[10] Lamontagne F, Agoritsas T, Siemieniuk R, Rochwerg B, Bartoszko J, Askie L, Macdonald H, Amin W, Bausch FJ, Burhan E, et al. A living WHO guideline on drugs to prevent covid-19. BMJ. 2021;372n526. 10.1136/bmj.n526. 33649077

[11] Working Group on Guidelines for T, Management of Anticoagulation in Hospitalized Patients with C, Pulmonary E, Pulmonary Vascular Diseases Group of the Chinese, Thoracic S, Pulmonary E, Pulmonary Vascular Disease Working Committee of Chinese Association of Chest P, National Cooperation Group on P, Treatment of Pulmonary E, Pulmonary Vascular D et al. National Program Office for Prevention Treatment of Pulmonary E: [Practice guidelines of thromboprophylaxis and management of anticoagulation in hospitalized patients with COVID-19]. *Zhonghua Yi Xue Za Zhi* 2023, 103:1–23.10.3760/cma.j.cn112137-2023-01-20-00115. 10.3760/cma.j.cn112137-2023-01-20-00115 36776125

[12] Di Micco P, Imbalzano E, Russo V, Attena E, Mandaliti V, Orlando L, Lombardi M, Di Micco G, Camporese G, Annunziata S, et al. Heparin and SARS-CoV-2: multiple pathophysiological links. Viruses. 2021;13(12). 10.3390/v13122486. 10.3390/v13122486 PMC8705068 34960754

[13] Goligher EC, Bradbury CA, McVerry BJ, Lawler PR, Berger JS, Gong MN, Carrier M, Reynolds HR, Kumar A, Turgeon AF (2021). Therapeutic anticoagulation with heparin in critically ill patients with Covid-19. N Engl J Med.

[14] Vedovati MC, Graziani M, Agnelli G, Becattini C (2023). Efficacy and safety of two heparin regimens for prevention of venous thromboembolism in hospitalized patients with COVID-19: a meta-analysis. Intern Emerg Med.

[15] Bohula EA, Berg DD, Lopes MS, Connors JM, Babar I, Barnett CF, Chaudhry SP, Chopra A, Ginete W, Ieong MH (2022). Anticoagulation and Antiplatelet Therapy for Prevention of Venous and arterial thrombotic events in critically ill patients with COVID-19: COVID-PACT. Circulation.

[16] Spyropoulos AC, Goldin M, Giannis D, Diab W, Wang J, Khanijo S, Mignatti A, Gianos E, Cohen M, Sharifova G (2021). Efficacy and safety of therapeutic-dose heparin vs Standard Prophylactic or Intermediate-Dose Heparins for Thromboprophylaxis in High-risk hospitalized patients with COVID-19: the HEP-COVID randomized clinical trial. JAMA Intern Med.

[17] Bonfim L, Guerini IS, Zambon MG, Pires GB, Silva ACF, Gobatto ALN, Lopes MA, Brosnahan SB. Optimal dosing of heparin for prophylactic anticoagulation in critically ill COVID-19 patients a systematic review and meta-analysis of randomized controlled trials. J Crit Care. 2023;77154344. 10.1016/j.jcrc.2023.154344. 10.1016/j.jcrc.2023.154344 PMC10211463 37244209

[18] Hess CN, Hsia J, Carroll IA, Nehler MR, Ruf W, Morrow DA, Nicolau JC, Berwanger O, Szarek M, Capell WH (2023). Novel tissue factor inhibition for Thromboprophylaxis in COVID-19: primary results of the ASPEN-COVID-19 Trial. Arterioscler Thromb Vasc Biol.

[19] Bargellini I, Cervelli R, Lunardi A, Scandiffio R, Daviddi F, Giorgi L, Cicorelli A, Crocetti L, Cioni R (2020). Spontaneous bleedings in COVID-19 patients: an emerging complication. Cardiovasc Intervent Radiol.

[20] Rostami M, Mansouritorghabeh H (2023). Significance of heparin induced thrombocytopenia (HIT) in COVID-19: a systematic review and meta-analysis. J Thromb Thrombolysis.

[21] Lannon M, Duda T, Greer A, Hewitt M, Sharma A, Martyniuk A, Owen J, Amin F, Sharma S (2023). Intracranial hemorrhage in patients treated for SARS-CoV-2 with extracorporeal membrane oxygenation: a systematic review and meta-analysis. J Crit Care.

[22] Rogani S, Calsolaro V, Franchi R, Calabrese AM, Okoye C, Monzani F. Spontaneous muscle hematoma in older patients with COVID-19: two case reports and literature review. BMC Geriatr. 2020;20(1):539. 10.1186/s12877-020-01963-4. 10.1186/s12877-020-01963-4 PMC7755066 33353545

[23] Patel I, Akoluk A, Douedi S, Upadhyaya V, Mazahir U, Costanzo E, Flynn D. Life-threatening Psoas Hematoma due to Retroperitoneal Hemorrhage in a COVID-19 patient on enoxaparin treated with arterial embolization: a Case Report. J Clin Med Res. 2020;12(7):458–61. 10.14740/jocmr4256. 10.14740/jocmr4256 PMC7331864 32655742

[24] Nakamura H, Ouchi G, Miyagi K, Higure Y, Otsuki M, Nishiyama N, Kinjo T, Nakamatsu M, Tateyama M, Kukita I, et al. Case Report: Iliopsoas Hematoma during the clinical course of severe COVID-19 in two male patients. Am J Trop Med Hyg. 2021;104(3):1018–21. 10.4269/ajtmh.20-1507. 10.4269/ajtmh.20-1507 PMC7941852 33534775

[25] Yeoh WC, Lee KT, Zainul NH, Syed Alwi SB, Low LL. Spontaneous retroperitoneal hematoma: a rare bleeding occurrence in COVID-19. Oxf Med Case Reports. 2021;2021(9):omab081. 10.1093/omcr/omab081. 10.1093/omcr/omab081 PMC8436278 34527254

[26] Kamijo H, Mochizuki K, Nakamura Y, Mori K, Ichikawa M, Nitta K, Imamura H. Nafamostat Mesylate Improved Survival outcomes of Sepsis patients who underwent blood purification: a Nationwide Registry Study in Japan. J Clin Med. 2020;9(8). 10.3390/jcm9082629. 10.3390/jcm9082629 PMC7464767 32823637

[27] Yamamoto M, Kiso M, Sakai-Tagawa Y, Iwatsuki-Horimoto K, Imai M, Takeda M, Kinoshita N, Ohmagari N, Gohda J, Semba K, et al. The Anticoagulant Nafamostat Potently inhibits SARS-CoV-2 S protein-mediated Fusion in a cell Fusion Assay System and viral infection in Vitro in a cell-type-dependent manner. Viruses. 2020;12(6). 10.3390/v12060629. PMC7354595 32532094

[28] Asakura H, Ogawa H (2020). Potential of heparin and nafamostat combination therapy for COVID-19. J Thromb Haemost.

[29] Jang S, Rhee JY (2020). Three cases of treatment with nafamostat in elderly patients with COVID-19 pneumonia who need oxygen therapy. Int J Infect Dis.

[30] Quinn TM, Gaughan EE, Bruce A, Antonelli J, O’Connor R, Li F, McNamara S, Koch O, MacKintosh C, Dockrell D et al. Randomised controlled trial of intravenous nafamostat mesylate in COVID pneumonitis: Phase 1b/2a experimental study to investigate safety, Pharmacokinetics and Pharmacodynamics. *EBioMedicine* 2022, 76:103856.10.1016/j.ebiom.2022.103856. 10.1016/j.ebiom.2022.103856 PMC8831100 35152152

[31] Buchtele N, Schörgenhofer C, Schwameis M, Jilma B, Schellongowski P, Herkner H, Riss K, Schmid M, Hermann A, Robak O (2022). Add-On prostaglandin E(1) in venovenous extracorporeal membrane oxygenation: a Randomized, Double-Blind, placebo-controlled pilot trial. Am J Respir Crit Care Med.

